# Trace Mineral Intake and Deficiencies in Older Adults Living in the Community and Institutions: A Systematic Review

**DOI:** 10.3390/nu12041072

**Published:** 2020-04-13

**Authors:** Zeynep Vural, Amanda Avery, Dimitris I. Kalogiros, Lisa J. Coneyworth, Simon J. M. Welham

**Affiliations:** 1 Nutrition and Dietetics, Division of Food, School of Biosciences, University of Nottingham, Sutton Bonington, Loughborough, Leicestershire LE12 5RD, UK; zeynep.vural1@nottingham.ac.uk (Z.V.); amanda.avery@nottingham.ac.uk (A.A.); lisa.coneyworth@nottingham.ac.uk (L.J.C.); 2 School of Mathematical Sciences, University of Nottingham, University Park, Nottingham NG7 2RD, UK; Dimitris.Kalogiros@nottingham.ac.uk

**Keywords:** Elderly, micronutrient, mineral, nutrition, iodine, zinc, selenium, iron, copper

## Abstract

The global population is ageing with many older adults suffering from age-related malnutrition, including micronutrient deficiencies. Adequate nutrient intake is vital to enable older adults to continue living independently and delay their institutionalisation, as well as to prevent deterioration of health status in those living in institutions. This systematic review investigated the insufficiency of trace minerals in older adults living independently and in institutions. We examined 28 studies following a cross-sectional or cohort design, including 7203 older adults (≥60) living independently in 13 Western countries and 2036 living in institutions in seven Western countries. The estimated average requirement (EAR) cut-off point method was used to calculate percentage insufficiency for eight trace minerals using extracted mean and standard deviation values. Zinc deficiency was observed in 31% of community-based women and 49% of men. This was higher for those in institutional care (50% and 66%, respectively). Selenium intakes were similarly compromised with deficiency in 49% women and 37% men in the community and 44% women and 27% men in institutions. We additionally found significant proportions of both populations showing insufficiency for iron, iodine and copper. This paper identifies consistent nutritional insufficiency for selenium, zinc, iodine and copper in older adults.

## 1. Introduction

The global population is now an ageing one, and the rate at which it is doing so is accelerating [1]. With life expectancy increasing, those aged 60 and over, who numbered 900 million in 2015, are expected to reach 2 billion by 2050 [1]. Nutrition is important to ensure people can maintain an active life, ageing in a healthy way [2]. However, older adults are prone to suffer from undernutrition due to decreased appetites, lack of hunger and a reduced food intake [3]. Undernutrition may lead to poor health outcomes, including frailty, functional deterioration, sarcopenia, immune dysfunction and morbidity [3]. The prevalence of age-related anorexia is around 25% in older adults living in the community and 85% among older adults living in nursing homes [4] and the prevalence of the risk of malnutrition has been reported as 27% and 48%, respectively [5]. Adequate nutritional intake and status plays a role in preventing adverse health outcomes and risk of institutionalisation, as well as delaying institutionalisation in older adults [6].

Micronutrient deficiencies (MNDs) generally originate due to the insufficient dietary intake of minerals and vitamins and are a feature of malnutrition [7]. MNDs and low dietary intakes among free-living older adults lead to functional decline, frailty and difficulties with independent living [8]. An increased prevalence of micronutrient deficiencies has been associated with an increased risk of frailty in community dwelling older women (HR 1.10; 95% CI, 1.01–1.20) [9]. A similar association was reported by Bartali et al. (2006) [10], who showed that inadequate dietary intakes of more than three nutrients in older adults aged 65 years and over is associated with frailty (OR: 2.12; 95% CI: 1.29–3.50). Trace minerals (iron, zinc, selenium, iodine, copper, chromium, manganese and molybdenum) perform vital functions within the body including thyroid metabolism, antioxidant activity and immune function. It is important to consider the impact of adequate trace mineral intakes and status on health, independence and delaying institutionalisation in older adults. To the best of our knowledge, this is the first systematic review that has focused on the dietary intakes of eight trace minerals in institutionalised and community dwelling older adults in Western countries.

The aim of this systematic review is to determine the dietary intake of eight trace minerals and any potential inadequacies within the older adult population at risk of such deficiency in both institutionalised and community settings in Western countries.

## 2. Materials and Methods

This systematic review was registered with the International Prospective Register of Systematic Reviews (PROSPERO) on 16 July 2019 (registration number CRD42019140923). The Preferred Items for Systematic Reviews and Meta-Analysis (PRISMA) guidelines were followed (Supplementary Figure S1).

### 2.1. Data Sources, Search Strategy and Selection Criteria

Electronic databases PUBMED, EMBASE and Web of Science were searched for studies published between 01/01/2006 and 14/06/2019. The complete list of search terms employed are presented in the supplementary material. National food consumption surveys were additionally searched and we also carried out manual searching of reference lists from relevant publications.

Full-text articles were transferred to a single database and duplicates removed. Titles and abstracts were scanned and considered eligible if they were of cross-sectional or cohort study design, included participants aged ≥60 years and reported mineral intake data. Full-text articles were evaluated by the authors using the inclusion and exclusion criteria detailed in Table 1. Institutionalised older adults were defined as dependent, living in institutions, including care homes, nursing homes, care centres and retirement homes. Community-dwelling older adults were defined as non-institutionalised, free-living in the community, at home.

### 2.2. Quality Assessment and Data Extraction

The Newcastle–Ottawa Scale and Cochrane coding manual for cohort studies were used to assess the quality of the included observational studies. These scales were combined by Ter Borg et al. [8], using the criteria applicable for observational studies (Supplementary Table S1). Subject selection bias was determined by assessing whether there is a predefined study population and whether there are inclusion and exclusion criteria. To address potential outcome bias, a studies’ quality was assessed according to whether there was a validated dietary assessment method and whether there was an assessment of selective reporting (presence or not). Studies were categorised as low, moderate or high quality according to awarded points of 0–2, 3–5, 5, respectively. Assessment criteria and points awarded the study quality are summarized in the supplementary material (Supplementary Table S2). For each study, author, publication year, study year, country, study design, quality score, participant characteristics (age, sample size by each sex), dietary assessment method and supplement usage were extracted (Table 2 and Table 3). Articles were double-checked if supplement intake was mentioned in the article to determine whether supplement intake was included in the dietary intake analysis. For each study, dietary intake of each trace mineral was extracted for each sex as mean and standard deviation (mean ± standard deviation; Table 4, Table 5, Table 6 and Table 7). If study data were presented in different subgroups by each sex (e.g., malnourished, at risk of malnutrition, well-nourished), pooled means and standard deviations were calculated. If the study was longitudinal, baseline data for each sex as mean and standard deviation was extracted; if baseline data were not available, follow-up data were included in the analysis.

### 2.3. Data Analysis

All analyses were undertaken using IBM Statistical Package for the Social Sciences (SPSS) Statistics version 24.0 (The International Business Machines Corporation (IBM) Company, Armonk, New York, USA). GraphPad Prism version 8.1.2 (GraphPad Software, San Diego, California, USA) was used to produce figures.

Analysis was carried out as outlined in a previous study [8]. After determining the quality score of each study by each sex, sensitivity analysis was performed using one-way ANOVA comparing mean values of each study-quality subgroup (low, moderate, high) to determine whether there were group differences. If there were differences between groups, a post hoc test was carried out to determine which sub-groups differed significantly from others. A *p*-value less than 0.05 (*p* < 0.05) was taken to accept that there were significant differences by study-quality subgroups in terms of mean mineral intake.

The following calculations were employed to determine pooled mean, pooled standard deviation, pooled confidence intervals:

***Pooled mean***.

$$Pmean=\frac{\left(n1\times m1\right)+\left(n2\times m2\right)+\left(n3\times m3\right)+\ldots +\left(nk+mk\right)}{\left(n1+n2+n3+\ldots +mk\right)}$$

where P_mean_ = pool mean value, n = sample size of the study, m = mean value of the study),

***Pooled standard deviation***.

$$S\mathrm{pooled}=\sqrt{\frac{\left(n1\;-1\right)s1^{2}+\left(n2\;-1\right)s2^{2}+\ldots +\left(nk\;-1\right)sk^{2}}{n1\;+n2\;+\ldots +nk\;-k}}$$

where S_pooled_ = pooled standard value, n = sample size of the individual study, S = standard deviation of the individual study),

***Pooled confidence interval***.

$$P_{\mathrm{CI}}=p\;\pm \;z*\;\sqrt{\frac{p\left(1-p\right)}{n}}$$

where PCI = pool confidence interval, p = percentage of deficient population, n = sample size, z = 1.96 for the 95% CI),

Pooled percentage below EAR as well as each individual studies’ percentage value below the EAR were calculated by each sex for each trace mineral.

Estimated average requirements (EAR) from the Institute of Medicine (IOM) were used for iron, zinc, selenium, iodine, copper and molybdenum but not for chromium and manganese. Given the evidence for the EAR value for chromium and manganese is lacking, these were excluded from the EAR cut point analysis. For chromium and manganese, the mean intake was compared with adequate intake to allow qualitative comparison. Recommendations based on sex-specific and age-specific EAR value (≥60 years) were used. To calculate the prevalence of insufficient intake for each mineral by each sex, the EAR cut-point method was used [38]. Symmetrical distribution and independency of both population intakes and recommendations are required to apply this method. The only datasets known to not be symmetrical are those for iron intake in menstruating women and protein intake in men [38]. Our data did not include either of these groups, so we adopted the assumptions made by the IOM and assumed symmetrical datasets in our study.

The percentage of insufficient mineral intakes in the study population was calculated as the proportion of the group with intakes below the estimated average requirement. In this method, mean and standard deviation, if normally distributed, can be used to calculate the percentage of the population with intakes below the recommendation and determine the risk for insufficiency.

The probability of inadequacy and percentage showing inadequacy of a population for each mineral with reference to its respective EAR were calculated using the formulae below.

***Probability of population inadequacy***.

(1) $$z=\frac{\left(x-µ\right)}{SD}$$

where x = estimated average requirement for each trace mineral, µ = mean intake of the study population, SD = standard deviation of the study population and z = probability score [39]. From this formula, the given probability (z value) was expressed as a percentage from a z probability table.

***Pooled percentage value showing the potential inadequacy***.

(2) $$Pp=\frac{\left(n1*p1\right)+\left(n2*p2\right)+\left(n3*p3\right)+\ldots +\left(nk+pk\right)}{n1+n2+n3+\ldots +nk}$$

where P_p_ = pooled percentage of deficiency, n = sample size of the each individual study and p = percentage of potential inadequacy from each study. If the deficiency in the pool was ≥20% in both sexes, it was accepted as a potential dietary concern.

## 3. Results

Four hundred and twenty-six papers were identified for the full-text assessment (Supplementary Figure S1). A total of 28 studies met the inclusion criteria (16 sampling free-living and 12 sampling institutionalised older adults), including 7203 (67% female) older adults living in the community from 13 Western countries, and 2036 (68% female) older adults living in institutions from seven Western countries (Table 2 and Table 3). Articles were grouped according to the quality of the study. Of the 16 studies concerning community-dwelling older adults, three fulfilled criteria constituting high quality and for the 12 studies relating to institutionalised people, one study met the “high quality” criteria (Supplementary Material Table S1). The results of the sensitivity analysis showed no significant group differences between the two different study quality groups in terms of mean trace mineral intake, which facilitated an EAR cut-point analysis from the mean and standard deviation of each study’s dietary trace mineral intake data by sex (Table 4, Table 5, Table 6 and Table 7).

### 3.1. Inadequacies of Older Adults Living in the Community

Available data regarding the mean dietary intake of iron, zinc, selenium, iodine, copper, chromium, manganese and molybdenum in older adults living in the community are shown in Table 4, Table 5 and Table 8. Several populations were found to be deficient for iron, zinc, selenium, iodine and copper.

#### 3.1.1. Iron

The number of studies finding >10% individuals consuming below the EAR were proportionally fewer for women (5 of 14) than men (6 of 11). When combining the data from all studies, the population consumed approximately twice the EAR (from 4710 women and 2346 men) with 11% of women and 13% men below the EAR.

#### 3.1.2. Zinc

Zinc consumption was as widely studied as iron, providing a pooled participant number of 4038 for women and 2282 for men. Of the 11 studies which examined zinc intake in females, 10 of them found that deficiency was present in >10% of the population, with seven showing >25% and five indicating deficiency in >40% of the female population. In males, 10 studies examined zinc intake. Of these, all showed deficiency in >10% of the population, with six studies suggesting that >50% males were deficient. Overall, 31% of females measured in all studies and 49% of males were found to consume below the EAR (Table 8; Supplementary Figure S2).

#### 3.1.3. Selenium

Selenium intake was measured in five studies for both women and men. In all studies, >25% of women were below EAR and this was found in four of the five studies including men, also. Several studies showed very low intakes for women and men. From all studies combined, average intake was greater than the EAR, but with 49% of women and 37% of men falling below the required intake levels.

#### 3.1.4. Iodine

Iodine was examined in only five of the studies included, three for women and two for men, with total participant numbers of 695 and 514, respectively. Approximately 22% of women were found to be consuming less than the EAR, whilst this only reached 10% for men.

#### 3.1.5. Copper

Copper consumption met requirements for most individuals, but there still remained a considerable number of women (27%) and men (21%) who were considered to be deficient.

Funnel plots were generated for each mineral (Supplementary Figure S3) in order to assess the degree of reporting bias. We plotted the proportion reported as deficient against study population size. For many of the plots, there was a relatively poor achievement of confinement within confidence boundaries, suggesting that the study populations chosen did not represent the wider population for whom these studies were relevant. Some degree of bias was detected for studies examining iron intake and a significant proportion which measured zinc intake fell outside of the confidence limits. This was particularly apparent for female data. For studies examining selenium, iodine and copper, it was apparent that, although in some instances the study data did conform to expectations, the number of studies were too few to make any definitive assessment of bias or lack of it.

### 3.2. Significant Levels of Inadequacy in Older Adults Living in Institutions

The number of individuals in institutions for whom mineral intake data was available, were considerably fewer than for those living in the community. For iron, zinc, selenium iodine and copper, the proportion of participants included were 27%, 23%, 38%, 37% and 24%, respectively, of the individuals measured in the community. The level of inadequacy for iron, selenium and copper, was not greatly different to that seen for community living participants. However, studies looking at zinc and in particular, iodine, showed a considerable decline in the level of intake in institutionalised individuals compared with community living participants (Table 9; Figure S2).

#### 3.2.1. Iron

The mean iron intake in women across all studies was only 62% of that for community living participants, however the majority of women in institutions (91%) were considered to be adequate for consumption levels, slightly higher than for community dwellers (89%). For men, the levels of intake were similar to those from the community with a similar number considered deficient (10% vs. 13%).

#### 3.2.2. Copper

The levels of copper intake in institutionalised participants were similar to those seen for community living participants and levels of inadequacy were also similar for women (27%) and men (25%).

#### 3.2.3. Selenium

Selenium intake was also similar amongst institution-based participants compared with their community dwelling counterparts. Deficient intake was again seen to be far more frequent in women (44%) than in men (27%).

#### 3.2.4. Zinc

The mean level of zinc intake for people living in institutions was on the limit of the EAR for women (7 mg day^−1^; EAR, 6.8 mg day^−1^) whilst for men, it was below the required levels (8.5 mg day^−1^; EAR, 9.4 mg day^−1^). Approximately 50% of women and 66% of men fell below the required intake level. For women, five of the nine studies included showed >50% inadequacy with two at >70%, whilst for men, all but one study (eight out of nine) showed >50% inadequacy with a couple of studies indicating deficiency in 95% of the population studied (Table 7).

#### 3.2.5. Iodine

The studies which considered iodine intake in institutionalised individuals, whilst few, indicated an even more severe impact than for other minerals (Table 6, Table 7 and Table 9; Figure S2). The mean intake for women was 42% and for men, 39% of the level seen for their counterparts living in the community. Inadequate iodine intake was observed in 78% of women and 67% of men studied with mean intake levels providing only 61% and 72% of the EAR for women and men, respectively.

Funnel plots for institution based studies showed similar findings to those seen for the community studies (Supplementary Figure S4). The studies examining iron intake appeared to be better contained within confidence boundaries and spread relatively evenly, indicating less bias in these observations than was seen for community based studies. This was also the case for zinc focused studies. However, those looking at selenium, iodine and copper were few and conclusions regarding bias could not be confidently drawn.

Hazard ratios (Supplementary Figure S5) indicated that risk of deficiency for iron, zinc and copper was similar for females and males living in both settings and that living in institutional care did not alter the risk of deficiency for these minerals. The risk of selenium deficiency in institutional populations was higher for females than for males and females were also shown to be more at risk of iodine deficiency in community dwelling populations, however, the differences between all of the groups were relatively small.

## 4. Discussion

This review identified the potential dietary insufficiency of four out of the eight trace minerals examined: (1) selenium; (2) zinc; (3) copper; and (4) iodine. There is a risk of insufficient dietary intake of selenium, copper and zinc from food sources for both genders, both for those living in the community and those in institutions. Whilst, older adults of both genders living in institutions are considered at risk of a potential inadequacy of iodine, among those living in the community, only women were found to be at risk of deficiency.

Selenium and iodine inadequacy in the European population of older adults (aged over 64) has been reported as being potentially over 21% by using the EAR cut-off point method [39]. Furthermore, the potential insufficiency of selenium and iodine has been reported to exceed 20% in relation to the EAR cut-off point method, for community living older adults (≥65 years) in Western countries [8].

### 4.1. Selenium

Selenium is a trace mineral required for a range of processes including antioxidant activity and conversion of thyroxine to the more active tri-iodothyronine [40]. Selenium forms the active centre of several enzymes involved in redox reactions, which protect the cell from oxidative damage. As a result, selenium plays a significant role in immune function and the prevention of cancer [41]. Reduced selenium status in older adults has been suggested to cause depletion of erythrocytes through oxidative stress resulting in anaemia [42]. Further observations of frailty with advancing age, assumed to be mediated via excess oxidative damage, are directly associated with low selenium status [43,44].

The current review identified a potential inadequacy of selenium in both genders and among both populations. Dietary inadequacy amongst older adults living in the community was found to be 49% for women and 37% for men and 44% and 27%, respectively, among those who were institutionalised. A study in New Zealand of 578 older adults aged eighty and above, and living in the community, revealed inadequate dietary intake of selenium in relation to the EAR value (i.e., over two-thirds of participants did not meet this value) [45]. The study employed two 24-h multiple-pass recalls as a dietary assessment method, in order to determine the selenium intake of the study population. However, this study relied on memory for its dietary assessment method. Therefore, the existence of any cognitive impairment in such an older age group could have had a considerable impact on the study results due to the memory based dietary assessment method, and the prevalence of deficiency may be overestimated. A further study, undertaken in Denmark, reported a higher percentage of inadequate dietary selenium among older adults aged between 65 and 81 living in the community. The assessment method consisted of three days of weighed diary intake, in order to determine the participants’ dietary intake of selenium. The participants did not take any dietary supplements [46]. The study found that 25% of men and 33% of women had a lower dietary intake than EAR. However, over 60% of older Danish men, and 80% of older Danish women, are known to use supplements [46], which could have led to an overestimation of micronutrient insufficiency.

Johnson and Begum [47] reported the inadequacy of the dietary selenium intake of institutionalised older adults in Canada, comparing it with the values recommended by the dietary reference intake value. They employed a 24-h recall dietary assessment method with 98 frail older adults, aged 65 and older. The potential reason for such inadequacy could be seen as institutionalisation and frailty, which are associated with a higher nutritional risk and a lower dietary intake of nutrients [48].

### 4.2. Iodine

Iodine is an essential mineral for the production of thyroid hormones. Deficiency can lead to thyroid dysfunction [49] which, in older adults, is a risk factor for the development of hyperthyroidism atrial fibrillation, accompanied by decreased cardiac function and embolism [50,51]. In addition, thyroid dysfunction has been reported to be associated in older adults with: (1) frailty; (2) neuromuscular dysfunction; (3) cognitive decline; and (4) muscle wasting; (5) osteoporosis; and (6) lipid abnormalities with atherosclerosis [52,53].

This review identified a potential insufficiency of iodine among institutionalised older adults of both genders, i.e., 78% of women and 67% of men. In addition, this was also found in 22% of older women living in the community. Further to these findings, a study of dietary iodine inadequacy among older Danish adults aged between 65 and 81 and living in the community [46], found that 28% of men and 46% of women had a lower dietary iodine intake than EAR. Over 90% of ingested iodine is excreted in urine within twenty-four hours [54], so urinary iodine excretion (UIE) therefore forms an effective marker to determine recent dietary intake. A recent UK national survey reported a median UIC of older people living in the community aged 65 and over as adequate, with 133 μg L^−1^ for men and 135 μg L^−1^ for women. However, other studies have reported mild and moderate iodine deficiencies. A study of 309 older adults (with a median age of eighty-nine) assessed the iodine status of people living in long-term residential care in New Zealand. Median UIC from spot urine samples was reported as 72 μg L^−1^, showing a mild iodine deficiency. Following the implementation of a bread fortification programme, along with iodized salt, 29% of the inhabitants were found to have an iodine concentration of less than 50 μg L^−1^ [49]. A further study by Buchanan et al. [55] reported a mild iodine deficiency in eighty-four older adults, aged between 60 and 95, living in care facilities in Australia. The median UIC was determined from three repeated fasting urine samples and was reported to be 71 μg L^−1^, i.e., showing mild iodine deficiency. A study undertaken by Olmedo Carrillo et al. [56] of 227 older adults in Spain, who were aged over 65, measured their mean urinary iodine concentration (UIC) by using the first-morning spot urine sample. The mean UIC of these older adults was reported as 109.33 μg L^−1^, with a confidence interval of 96.75–121.50 μg L^−1^. A further study of 189 menopausal women in Italy, aged between 51 and 86, used morning spot urine samples to assess median UIC [52], which was reported to be 30 μg L^−1^, i.e., indicating moderate iodine deficiency.

Studies assessing iodine intake and status primarily focus on younger adults, resulting in limited data being available for older adults [52]. This was also true of our pooled analysis, which contained only three eligible studies of older adults living in the community and two studies of those living in institutions. Even though focusing studies on younger age groups instead of older adults are more common, it should be considered that the correction of iodine deficiency in older adults could help to decrease thyroid autonomy processes [52] which is common in geographic areas with iodine deficiency [57]. There is also an association between higher rates of hyperthyroidism (which commonly occur in mild to moderate iodine-deficient areas) and the incidence of toxic multinodular goitre. This is due to the growth of autonomous thyrocytes being promoted by iodine deficiency. Under conditions of mild to moderate iodine deficiency, to compensate for low iodine intake and to maintain normal thyroid gland function, thyroid activity increases and this thyroid stimulation causes the higher rates of hyperthyroidism and toxic nodular goitre. Thyroid activity becomes normalised and thus nodular autonomy is reduced in the correction of iodine deficiency [58]. A decrease of this autonomization process through the correction of iodine deficiency may consequently help to prevent the cardiovascular and skeletal results of hyperthyroidism in this older population [52].

### 4.3. Zinc

Zinc deficiency causes dysregulation of immune function, resulting in increased levels of inflammatory cytokines (i.e., interleukin-6 and tumour necrosis factor α), alongside deterioration of the antioxidant protection of cells and an increase in susceptibility to infection [59]. Dietary zinc deficiency is also associated with increased oxidative stress, playing a role in determining the pathologies of cardiovascular diseases in the elderly. Furthermore, zinc deficiency is associated with osteoporosis due to zinc’s structural role in the bone matrix mediating bone deposition and resorption [60].

We identified a potential inadequacy of zinc among both genders and in both population groups covered in the current review. This was found to be 31% for women and 49% for men among those dwelling in the community and 50% for women and 66% for men among institutionalised older adults. Our findings support observations by others showing zinc deficiency among older adults living in the community [61] and institutions [47]. A further study of 632 long-term care residents in Canada, who were aged 65 and over, also reported a dietary inadequacy of zinc [62].

### 4.4. Copper

Copper is an antioxidant trace mineral and a cofactor for several enzymes involved in cell oxidation, while also playing a significant role in immune function. Low dietary intake of copper has been found associated with a decreased immune response in older adults [63], along with an increased risk of ischemic heart disease [64].

We identified a potential inadequacy of dietary copper solely from food sources in both genders and in both population groups examined by the present review.

As seen in this review, dietary inadequacy of trace minerals (selenium, zinc, copper and iodine) emerge as a significant health issue in older adults considering the roles of these trace minerals in mediating antioxidant activity, thyroid function, immune response and cardiovascular health. The reasons behind mineral deficiencies are multifactorial. Older adults tend to consume less food, which causes an inadequate intake of minerals, which is the common reason underlying deficiency [65]. Several factors including social, economic, psychological and physiological have an impact on decreased food consumption which may cause inadequate mineral intake [66,67]. Reduced appetite resulting from a decrease in chewing and swallowing efficiency, taste and smell function, saliva production and poor oral and dental health, as well as changes in the digestive tract are associated with decreased food consumption in later life [68,69]. In addition, long-standing illnesses including diabetes, hypertension, and kidney disease requiring dietary restriction may limit food choice in older adults [70,71]. Additionally, common medications causing the reduction in appetite may contribute to inadequate intake of minerals [72]. Physical disability including poor mobility which restricts access to food sources, their procurement and preparation [73] and a decrease in physical balance and strength, along with limitations in manual dexterity also have a negative impact on food intake by affecting food procuration and cooking [74]. This reduction in food intake results in insufficient intake of minerals. Social and psychological changes with ageing may negatively impact food consumption and, thus, result in inadequacy of mineral intake. Lack of motivation to eat food, depression, stress [75], cognitive decline with ageing resulting in a decrease in food consumption due to forgetfulness and confusion [75], living arrangements (living alone or family, living in community or institutions), socialization, isolation from society and loneliness (eating alone or others), bereavement and grief [76] are other potential factors in later life. In addition to these factors, lower socioeconomic status including lower-income and lower education level [77] as well as limited knowledge and skills associated with nutrition [73] have an impact on this food reduction, leading to inadequate mineral intake.

Furthermore, another significant factor leading to an inadequate intake of minerals is the mineral content of foods eaten [78]. Food processing including preparation, cooking practices [79], the environment and soil where plants are grown have a significant role in determining mineral contents of foods consumed [78].

Undoubtedly, reasons mentioned above have a significant impact on the inadequate intake of minerals. However, even if there is an adequate dietary intake of minerals, several factors can impact on mineral status and cause deficiency, including: (1) malabsorption disorders; (2) use of medication; (3) amount and type of micronutrients taken; (4) interactions with other dietary nutrients; and (5) supplementation. Secondly, insufficient mineral intake can be improved by the appropriate use of supplements, whilst exercising caution to prevent overconsumption. For example, data from a Dutch National Food consumption survey of older adults living in the community, aged 70 and over, revealed that supplements of trace minerals (i.e., copper, selenium, iodine and zinc) alongside additional fortification of iodine, contributed to the total intake of these minerals and decreased the number of those individuals falling below the average requirement [21]. Similarly, the use of zinc supplementation in the USA was found to significantly improve nutrient intake, while at the same time reducing the proportion of the population below EAR, when compared to non-supplement users [80].

Dietary intake of trace minerals from food sources alone forms only part of the total mineral intake, particularly taking into account the increasing prevalence of older adults using vitamin and mineral supplements [81]. Although studies in the USA have demonstrated an increase in the use of mineral and vitamin supplements, this remains more common in older than younger adults [82,83,84]. Only a limited number of studies have been undertaken in Europe to determine the prevalence of dietary supplementation [85]. A KORA-Age study undertaken in Germany in 2009, on 1079 older adults aged 65 and over, reported the prevalence of dietary supplementation, i.e., 54.3% in women and 33.8% in men [85]. This was also reported among 40% of adults in Canada aged >51 [86]. In the USA, 70% of older adults aged over seventy-one were found to use dietary supplements [82].

The above discussion leads to the conclusion that, due to common usage of supplements in older adults, it is important to determine the total picture of mineral exposure, i.e., mineral intake from both food sources and supplements as well as supplements’ type and amount [82].

## 5. Strengths and Limitations of the Study

This current review contains a number of strengths and limitations. One of its most significant limitations is the need to interpret individual studies employing different dietary assessment methods. Such methods have several advantages and disadvantages. For example, being subject to memory recall (which is, by nature, subjective) [87], can impact on the reliability of the resulting data and, thus, the calculations of any potential inadequacy in the pooled analysis.

Furthermore, food composition tables and software developed for use in specific countries can influence the dietary intake calculation of individual studies and thus the pooled analyses. A further limitation concerns the lack of any standard nutrient recommendations, which can therefore change from country to country, resulting in different dietary national nutrient recommendations, causing confusion and reducing homogenisation between studies. For example, WHO/FAO, EC and 22 European countries have their own nutrient recommendations [88], as well as the recommendations from other Western countries, including IOM.

These different nutrient recommendations impact on the estimation of potential inadequacy among the study population. For instance, the present review has employed IOM recommendations to calculate the percentage of any potential inadequacy amongst older adults. The EAR value of zinc is 6.8 and 9.4 mg/day for older adults. In accordance with this recommendation, the study results estimated a potential risk of zinc inadequacy. However, if, for example, we had instead used the Nordic Nutrition recommendations, the percentage of the population found to be at risk of inadequate intake would be lower, due to Nordic recommendations concerning zinc being 5 mg per day for women and 6 mg for men. Therefore, the selected recommendations can have a significant impact on the calculations determining any potential prevalence.

In addition, the limited number of studies employed in this study could potentially have a considerable impact on the results. In relation to iodine, only two studies meeting the inclusion and exclusion criteria for both men and women among institutionalised older adults have been included. This is a relatively low number of studies and thus a high inadequacy in one study may have had a direct impact on the total result. A limited number of studies were also used to examine copper and selenium, with the results potentially shaped by an assumed inadequacy or adequacy from only one study, and thus the total results could be called into question.

Funnel plots (Supplementary Figures S3 and S4) showed variable findings depending on the mineral studied and the lifestyle context. There was evidence of differing degrees of reporting bias in both the community setting and in institutions, although for some, this was less apparent (e.g., iron in institutional populations; Supplementary Figure S4). The overall suggestion of potential bias implies a lack of studies providing sufficient information for all populations. This may be a consequence of the sheer lack of relevant studies, or potentially may reflect the propensity for studies showing negative values not being published. This would argue for a need for more studies to be conducted in all settings to enable generation of a reliable set of data to help provide informed dietary guidance.

A further potential limitation concerns the interpretation of study results, due to the potential use of supplements. We excluded studies including an intake of supplements from the dietary intake analysis, in order to determine dietary mineral intake from the food source alone. This was due to studies including supplements in the dietary intake analysis lacking clarity when it comes to the amount and type of supplement employed. The exclusion of such studies can be considered a strength of the present research, but there is also a need to recognise that the use of supplements is prevalent in an ageing population. Thus, even if there is a fairly high level of dietary inadequacy from food sources, this can be compensated by supplements. This results in difficulties in generalizing study results as the total picture of micronutrient inadequacies in an ageing population, particularly in the absence of information concerning the prevalence, amount and type of supplement usage.

In addition, we made assumptions concerning the normal distribution of usual intakes and dietary requirement and accepted all symmetric, because the EAR cut-point method requires symmetric distribution of the requirements and usual intake. However, this assumption may result in a biased estimate, i.e., an over- or underestimation of the prevalence of insufficiency [38].

This review has revealed the limited study data available from Western countries showing the dietary intake of trace minerals from food sources alone, particularly in relation to older adults aged 65 and over, and specifically for those living in institutions. It has only included recent studies, due to dietary habits, consumption trends and policies relating to food fortification policies (i.e., iodised salt and iodine fortification of bread or milk) potentially changing over time. In addition, this review focused on studies undertaken in Western countries, in order to give more homogenised results. The strengths of this paper include the provision of a general picture of the total dietary intake of trace minerals from food sources.

Despite this review establishing a general picture of the dietary intake of trace minerals from food sources alone, along with the potential risk of inadequacy in the population, it is not possible for these findings alone to confirm the existence of trace mineral deficiency. This is due to deficiency classification depending on several factors, including: (1) the dietary intake of minerals; (2) their absorption and metabolism; (3) the impact of medication and medical conditions; (4) the use of supplements; and (5) the cut-off points employed to describe mineral deficiencies.

## 6. Conclusions

This review examined the dietary intake and potential deficiency of eight trace minerals in older adults, including those living in the community and those in institutions. Four of the eight trace minerals (i.e., selenium, zinc, iodine and copper) were found to be of potential concern, due to a high prevalence of insufficiency which, for several minerals, was worsened by the requirement for institutional care. This study concludes that, even if these results are unable to determine the exact levels of dietary mineral deficiency among older adults, it can offer a general picture of the potential insufficiency of trace minerals from foods among this population. We provide strong support for the more detailed monitoring of mineral nutrition within elderly populations, in particular, those living in institutions. The findings should be considered within population nutritional health strategies which aim to improve the nutritional status of older adults with a pragmatic public health approach such as the provision of menus providing the minerals identified as being at risk, the use of iodised salt in cooking in all care home settings and/or the recommendation that older people should be taking a multi-mineral supplement.

## Figures and Tables

**Table 1:** Summary of the inclusion and exclusion criteria.

Inclusion Criteria	Exclusion Criteria
Studies reporting dietary intake of at least one trace minerals as mean and standard deviation	Studies including supplement intake as a part of the dietary intake data analysis
Studies including community dwelling or institutionalized older adults aged 60 and over	Studies including enteral parenteral feeding data as well as adjusted data
Studies having cross sectional or cohort study design	Studies including overall (both sexes together) trace mineral intake data
Studies clearly defined dietary intake method and coming from Western countries	Studies including hospitalized patients
Full text articles published in English language	

**Table 2:** Summary of the included studies, evaluating trace mineral intake in older adults living in the community.

Participant Characteristics								Supplement Intake	
Author	Study Year	Country	Study Design	Quality Score	Age (Years)	Subjects (n)	Dietary Assessment Method	Reported	Included in the Analysis
(Wyka et al. [11])	NA	Poland	Cross-sectional	Moderate	≥60	174 Female 64 Male	24HR	Not mentioned	Not mentioned
(Zhu et al. [12])	NA	Australia	Longitudinal	High	70–85	911 female	FFQ	Excluded	No
(Jiménez-Redondo et al. [13])	2011	Spain	Cross-sectional	Moderate	≥80	53 female 30 male	24HR	Not mentioned	Not mentioned
(Engelheart and Akner. [14])	2002–2010	Sweden	Observational	Moderate	64–100	84 Female 52 Male	3-4 d DR	Excluded	No
(Roussel et al. [15])	NA	France	Cross-sectional	Moderate	70–85	8 Female 4 Male	3 d DR	Excluded	No
(Dumartheray et al. [16])	2004	Switzerland	Prospective	Moderate	75–87	401 Female	FFQ	Not mentioned	Not mentioned
(Li et al. [17])	2014–2015	USA	Cross-sectional	Moderate	≥65	97 Female	3×24HR	Not mentioned	Not mentioned
(Destefani et al. [18])	NA	Brazil	Cross-sectional	Moderate	≥60	135 Female	2×24HR	Not mentioned	Not mentioned
(Feart et al. [19])	2001–2002	France	Prospective	Moderate	≥65	988 Female, 607 Male	24HR-FFQ	Not mentioned	Not mentioned
(Martínez Tomé et al. [20])	NA	Spain	Cross-sectional	Moderate	65–89	117 Female, 83 Male	2×24HR	Not mentioned	Not mentioned
(Ocke et al. [21])	2010–2012	The Netherlands	National Survey	High	≥70	366 Female, 373 Male	2×24HR	Yes	No
(Sette et al. [22])	2005–2006	Italy	National Survey	Moderate	≥65	316 Female, 202 Male	3 d DR	Yes	No
(Biró et al. [23])	2009	Hungary	National Survey	Moderate	>60	475 Female, 270 Male	3 d DR	Yes	No
(National Diet and Nutrition Survey [24])	2014–2016	UK	National Survey	Moderate	≥65	194 Female, 141 Male	4 d DR	Yes	No
(USDA et al. [25])	2015–2016	USA	National Survey	High	≥70	414 Female, 418 Male	2×24HR	Excluded	No
(NANS [26])	2008–2010	Ireland	National Survey	Moderate	≥65	120 Female, 106 Male	4 d DR	YES	No

24HR = 24 h dietary recall, FFQ = Food Frequency Questionnaire, DR = Dietary Record, NA = Not applicable, USDA = US Department of Agriculture.

**Table 3:** Summary of the included studies, evaluating trace mineral intake in older adults living in the institutions.

								Supplement Intake	
Author	Study Year	Country	Study Design	Quality Score	Age (Years)	Subjects (n)	Dietary Assessment Method	Reported	Included in the Analysis
(González et al. [27])	NA	Spain	Cross-sectional	Moderate	60–80	125 Female, 80 Male	FFQ	Not mentioned	Not mentioned
(Rakıcıoğlu et al. [28])	2007–2009	Turkey	Longitudinal study	Moderate	≥65	45 Female, 57 Male	24HR	Not mentioned	Not mentioned
(Fernández-Barrés et al. [29])	NA	Spain	Cross-sectional	High	≥65	128 Female, 62 Male	FFQ	Excluded	No
(Woods et al. [30])	NA	Australia	Cross-sectional	Moderate	≥65	72 Female, 23 Male	3 d weighed DR	Yes	No
(Iuliano et al. [31])	NA	Australia	Cross-sectional	Moderate	67–99	151 Female 48 Male	3–6 d weighed DR	Yes	Not included
(Lengyel et al. [32])	1999	Canada	Cross-sectional	Moderate	≥65	31 Female, 17 Male	3 d weighed DR	Not mentioned	Not mentioned
(Lopez-Contreras et al. [33])	NA	Spain	Cross-sectional	Moderate	65–96	151 Female, 101 Male	4 d weighed DR	Not mentioned	Not mentioned
(Leslie et al. [34])	2002–2003	UK	Cross-sectional	Moderate	84–100	21 Female, 14 Male	3 d weighed DR	Yes	Not included mineral intake analysis
(Aghdassi et al. [35])	1997–1999	Canada	Cross-sectional	Moderate	≥65	299 Female, 108 Male	3 d DR	Excluded	No
(Engelheart and Akner [14])	2002–2010	Sweden	Observational	Moderate	66–103	93 Female, 35 Male	3 d DR, 5 d weighed DR	Excluded	No
(Rodríguez-Rejón et al. [36])	2013–2016	Spain	Cross-sectional	Moderate	≥70	187 Female, 62 Male	7 d weighed DR	Not mentioned	Not mentioned
Assis et al. [37]	NA	Brazil	Cross-sectional	Moderate	≥60	157 Female, 59 Male	6 d weighed DR	Not mentioned	Not mentioned

24HR = 24 h dietary recall, FFQ = Food Frequency Questionnaire, DR = Dietary Record, NA = Not applicable.

**Table 4:** Percentage of free-living older women with mineral intakes below the Estimated Average Requirement (EAR) or Adequate Intake (AI) and therefore at increased risk for inadequacy. Mineral intakes are presented as mean + SD. Individual study date and percentage of the free-living population at risk for inadequacy, trace mineral intakes among older women.

Reference	Country	Subjects (n)	Iron (Mean ± SD) % EAR: 5 mg/day	Zinc (Mean ± SD) % EAR: 6.8 mg/day	Selenium (Mean ± SD) % EAR: 45 μg/day	Iodine (Mean ± SD) % EAR: 95 μg/day	Copper (Mean ± SD) % EAR: 0.7 mg/day	Molybdenum (Mean ± SD) % EAR: 34 μg/day	Chromium (Mean ± SD) AI: 20 μg/day	Manganese (Mean ± SD) AI: 1.8 mg/day
(Wyka et al. [11])	Poland	174	(7.2 ± 2.9) 23%	-	-	-	-		-	-
(Zhu et al. [12])	Australia	911	(12.3 ± 4.4) 5%	(10.6 ± 3.5) 14%	-	-	-	-	-	-
(Jiménez-Redondo et al. [13])	Spain	53	(9.3 ± 3.2) 9%	(7.2 ± 3.7) 46%	(62.3 ± 35.8) 32%	-	-	-	-	-
(Engelheart and Akner [14])	Sweden	84	(8 ± 2) 7%	(8 ± 2) 28%	(28 ± 10) 96%	-	-	-	-	-
(Roussel et al. [15])	France	8	-	-	-	-	-	-	(42.74 ± 14.67)	-
(Dumartheray et al. [16])	Swiss	401	(11.6 ± 3.7) 4%	-	-	-	-	-	-	-
(Li et al. [17])	USA	97	(11.3 ± 4.8) 10%	-	-	-	-	-	-	-
(Destefani et al. [18])	Brazil	135	-	-	-	(100.7 ± 39.2) 44%	-	-	-	-
(Feart et al. [19])	France	988	(9.7 ± 4.9) 17%	(7.7 ± 7.4) 45%	-	-	-	-	-	-
(Martínez Tomé et al. [20])	Spain	117	(18.6 ± 5.4) 1%	(11.9 ± 2.8) 4%	-	-	(1.5 ± 0.6) 9%	-	-	(3.4 ± 0.9)
(Ocke et al. [21])	The Netherlands	366	(9.1 ± 2.9) 8%	(9.7 ± 3.3) 19%	(42.1 ± 16.3) 57%	(146 ± 49) 15%	(1 ± 0.3) 16%	-	-	-
(Sette et al. [22])	Italy	316	(10.0 ± 3.0) 5%	(9.9 ± 2.9) 14%	-	-	-	-	-	-
(Biró et al. [23])	Hungary	475	(9.2 ± 2.4) 4%	(7.0 ± 1.9) 46%	-	-	(0.9 ± 0.4) 31%	-	(55.6 ± 23.0)	(2.2 ± 3.4)
(National Diet and Nutrition Survey [24])	UK	194	(8.4 ± 3.0) 13%	(7.1 ± 2.4) 45%	(38 ± 16) 67%	(147 ± 64) 21%	-	-	-	-
(USDA et al. [25])	USA	414	(11.5 ± 11.2) 28%	(8.2 ± 8.3) 43%	(84.5 ± 57.2) 25%		(1 ± 0.8) 35%	-	-	-
(NANS [26])	Ireland	120	(10 ± 3.7) 9%	(8 ± 2.6) 33%	-	-	(1. ± 0.7) 33%	-	-	(3.6 ± 1.9)
Pool Mean Pool STANDARD DEVIATION Pool PERCENTAGE BELOW EAR			10.5 5.6 11%	8.8 5.1 31%	57.1 37.7 49%	137.5 52 22%	1 0.6 27%	- - -	55.4 22.9 -	2.6 2.9 -

**EAR:** Estimated average requirement, **n:** Sample size, **%:** Potential inadequacy (percentage of population who below the estimated average requirement), AI: Adequate intake.

**Table 5:** Percentage of free-living older men with mineral intakes below the Estimated Average Requirement (EAR) or Adequate Intake (AI) and therefore at increased risk for inadequacy. Mineral intakes are presented as mean + SD. Individual study data and percentage of the free-living population at risk for inadequacy, trace mineral intakes among older men.

Reference	Country	Subject (n)	Iron (Mean ± SD) % EAR: 6 mg/day	Zinc (Mean ± SD) % EAR: 9.4 mg/day	Selenium (Mean ± SD) % EAR: 45 μg/day	Iodine (Mean ± SD) % EAR: 95 μg/day	Copper (Mean ± SD) % EAR: 0.7 mg/day	Molybdenum (Mean ± SD) % EAR: 34 μg/day	Chromium (Mean ± SD) AI: 30 μg/day	Manganese (Mean ± SD) AI: 2.3 mg/day
(Wyka et al. [11])	Poland	64	(10.3 ± 7) 27%	-	-	-	-	-	-	-
(Jiménez-Redondo et al. [13])	Spain	30	(10.8 ± 3.0) 5%	(7.3 ± 2.1) 84%	(76.5 ± 29.5) 14%	-	-	-	-	-
(Engelheart and Akner [14])	Sweden	52	(9 ± 3) 15%	(9 ± 2) 58%	(34 ± 13) 80%	-	-	-	-	-
(Roussel et al. [15])	France	4	-	-	-	-	-	-	(35.18 ± 10.88)	-
(Feart et al. [19])	France	607	(13.3 ± 6.2) 12%	(7.0 ± 5.3) 67%	-	-	-	-	-	-
(Martínez Tomé et al. [20])	Spain	83	(16.4 ± 5.3) 3%	(10.4 ± 3.3) 38%	-	-	(1.1 ± 0.4) 16%	-	-	(3.2 ± 1)
(Ocke et al. [21])	The Netherlands	373	(11.4 ± 4.2) 10%	(11.1 ± 3.6) 32%	(49 ± 21) 42%	(172.2 ± 55) 8%	(1.2 ± 0.5) 16%	-	-	-
(Sette et al. [22])	Italy	202	(13.2 ± 3.8) 3%	(12.2 ± 3.2) 19%	-	-	-		-	-
(Biró et al. [23])	Hungary	270	(11.1 ± 3.0) 4%	(8.8 ± 2.5) 59%	-	-	(1.1 ± 0.4) 16%	-	(59.5 ± 24.3)	(2.4 ± 0.8)
(National Diet and Nutrition Survey [24])	UK	141	(10.6 ± 3.3) 8%	(8.8 ± 2.7) 59%	(50 ± 2) 41%	(186 ± 84) 14%	-	-	-	-
(USDA et al. [25])	USA	418	(16.2 ± 19.8) 30%	(11.7 ± 11.7) 42%	(107.9 ± 104.9) 27%	-	(1.2 ± 1) 31%	-	-	-
(NANS [26])	Ireland	106	(11.7 ± 4.6) 11%	(9.4 ± 3.1) 50%	-	-	(1.1 ± 0.5) 21%		-	(4 ± 1)
MEAN (total)) STANDARD DEVIATION (total) PERCENTAGE BELOW EAR (total)			12.9 9.4 13%	9.6 6.1 49%	73.5 69.4 37%	176 64.2 10%	1.16 0.69 21%	- --	59.1 24.1 -	2.9 0.8 -

**EAR:** Estimated average requirement, **n:** Sample size, **%**: Potential inadequacy (percentage of population who below the estimated average requirement), AI: Adequate intake.

**Table 6:** Individual study data and percentage of institutionalised older adults at risk of inadequacy for trace minerals in women.

Reference	Country	Subjects (n)	Iron (Mean ± SD) % EAR: 5 mg/day	Zinc (Mean ± SD) % EAR: 6.8 mg/day	Selenium (Mean ± SD) % EAR: 45 μg/day	Iodine (Mean ± SD) % EAR: 95 μg/day	Copper (Mean ± SD) % EAR: 0.7 mg/day	Molybdenum (Mean ± SD) % EAR: 34 μg/day	Chromium (Mean ± SD) AI: 20 μg/day	Manganese (Mean ± SD) AI: 1.8 mg/day
(González et al. [27])	Spain	125	-	-	(94.4 ± 23.6) 25%	-	-	-	-	-
(Rakıcıoğlu et al. [28])	Turkey	45	(9.5 ± 4.0) 13%	(8.8 ± 3.8) 30%	-	-	-	-	-	-
(Fernández-Barrés et al. [29])	Spain	128	(6.9 ± 1.7) 13%	-	-	-	-	-	-	-
(Woods et al. [30])	Australia	72	(8.2 ± 1.9) 5%	(6.6 ± 1.3) 56%	-	-	-	-	-	-
(Iuliano et al. [31])	Australia	151	(7.7 ± 2.2) 11%	(7.1 ± 1.8) 43%	-	(92.1 ± 27.8) 54%	-	-	-	-
(Lengyel et al. [32]	Canada	31	(9.4 ± 2.7) 5%	(5.6 ± 2.3) 70%	-	-	-	-	-	-
(Lopez-Contreras et al. [33])	Spain	151	(11.5 ± 3.5) 3%	-	-	-	-	-	-	-
(Leslie et al. [34])	UK	21	-	(5.7 ± 1.4) 79%	-	-	-	-	-	-
(Aghdassi et al. [35])	Canada	299	(10.7 ± 3.6) 6%	(8.2 ± 2.7) 30%	-	-	(1.1 ± 0.5) 21%	-	-	-
(Engelheart and Akner [14])	Sweden	93	(6 ± 2) 31%	(7 ± 2) 46%	(27 ± 8) 98%	-	-	-	-	-
(Rodríguez-Rejón et al. [36])	Spain	187	(7.27 ± 1.78) 10%	(5.64 ± 1.78) 74%	(44.27 ± 20.24) 52%	(29.89 ± 28.72) 98%	(0.78 ± 0.23) 36%	-	-	-
(Assis et al. [37])	Brazil	157	(9.7 ± 2.33) 2%	(6.16 ± 1.95) 63%	(50.8 ± 18.19) 37%	-	-	-	-	-
MEAN (Pool) STANDARD DEVIATION (Pool) PERCENTAGE BELOW EAR (Pool)			6.5 2.7 9%	7 2.2 50%	54.4 19.1 44%	57.7 28.3 78%	0.98 0.4 27%	-	-	-

**EAR:** Estimated average requirement, **n:** Sample size, **%:** Potential inadequacy (percentage of population who below the estimated average requirement), AI: Adequate intake.

**Table 7:** Individual study data and percentage of institutionalised older adults at risk for inadequacy for trace minerals in men.

Reference	Country	Subject (n)	Iron (Mean ± SD) % EAR: 6 mg/day	Zinc (Mean ± SD) % EAR: 9.4 mg/day	Selenium (Mean ± SD) % EAR: 45 μg/day	Iodine (Mean ± SD) % EAR: 95 μg/day	Copper (Mean ± SD) % EAR: 0.7 mg/day	Molybdenum (Mean ± SD) % EAR: 34 μg/day	Chromium (Mean ± SD) AI: 30 μg/day	Manganese (Mean ± SD) AI: 2.3 mg/day
(González et al. [27])	Spain	80	-	-	(107.1 ± 32.2) 3%	-	-	-	-	-
(Rakıcıoğlu et al. [28])	Turkey	57	(12.5 ± 4.5) 7%	(11.2 ± 4.1) 33%	-	-	-	-	-	-
(Fernández-Barrés et al. [29])	Spain	62	(7.4 ± 2.5) 29%	-			-	-	-	-
(Woods et al. [30])	Australia	23	(10.8 ± 4.1) 12%	(8.7 ± 2.2) 63%	-	-	-	-	-	-
(Iuliano et al. [31])	Australia	48	(9.7 ± 3.9) 17%	(8.8 ± 2.5) 59%	-	(114.7 ± 34.1) 28%	-	-	-	-
(Lengyel et al. [32])	Canada	17	(12.2 ± 3.3) 3%	(7.5 ± 2.3) 80%	-	-	-	-	-	-
(Lopez-Contreras et al. [33])	Spain	101	(13.6 ± 4.4) 4%	-	-	-	-	-	-	-
(Leslie et al. [34])	UK	14	-	(6.2 ± 1.8) 96%	-	-	-	-	-	-
(Aghdassi et al. [35])	Canada	108	(11.1 ± 3.5) 7%	(8.5 ± 2.4) 65%	-	-	(1.1 ± 0.5) 21%	-	-	-
(Engelheart and Akner [14])	Sweden	35	(8 ± 2) 16%	(9 ± 3) 55%	(30 ± 9) 95%	-	-		-	-
(Rodríguez-Rejón et al. [36])	Spain	62	(8 ± 1.73) 12%	(6.35 ± 1.81) 95%	(51.78 ± 20.16) 37%	(32.66 ± 28.66) 98%	(0.8 ± 0.2) 31%	-	-	-
(Assis et al. [37])	Brazil	59	(12.52 ± 2.38) 0.3%	(8.44 ± 2.1) 68%	(70.59 ± 18) 8%	-	-	-	-	-
MEAN (total)) STANDARD DEVIATION (total) PERCENTAGE BELOW EAR (total)			10.8 11.9 10%	8.5 2.6 66%	72 23.5 27%	68.4 31.1 67%	0.99 0.17 25%	-	-	-

**EAR**: Estimated average requirement, **n:** Sample size **%:** Potential inadequacy (percentage of population who below the estimated average requirement), AI: Adequate intake.

**Table 8:** Daily trace mineral intake and percentage of inadequate intakes of older adults living in the community.

Nutrient	Sex	Studies (n)	Pooled (n)	Unit	EAR	Mean	SD	Percentage below EAR^*^	95% CI
Iron	W M	14 11	4710 2346	mg/d	5 6	10.5 12.9	5.6 9.4	11 13	10–12 12–14
Selenium	W M	5 5	1111 1014	μg/d	45 45	57.1 73.5	37.7 69.4	49 37	46–52 34–40
Zinc	W M	11 10	4038 2282	mg/d	6.8 9.4	8.8 9.6	5.1 6.1	31 49	30–32 47–51
Iodine	W M	3 2	695 514	μg/d	95 95	137.5 176	52 64.2	22 10	39–47 7–13
Copper	W M	5 5	1492 1250	mg/d	0.7 0.7	1 1.16	0.6 0.69	27 21	25–29 19–23
Molybdenum	W M	- -	- -	μg/d	34 34	- -	- -	- -	- -
Manganese	W M	3 3	712 459	mg/d mg/d	1.8 + 2.3 +	2.6 2.9	2.9 0.8	NA	-
Chromium	W M	2 2	483 274	μg/d μg/d	20 + 30 +	55.4 59.1	22.9 24.1	NA	-

EAR = Estimated Average Requirement, CI = Confidence Interval, *= Mean percentage of insufficient intake, calculated from EAR cut-point method, += Adequate Intake, not enable to apply EAR cut-point method, NA= Not applicable because mean intake is above the adequate intake, low prevalence of deficiency is assumed,–Not available study.

**Table 9:** Daily trace mineral intake and percentage of inadequate intakes of older adults living in institutions.

Nutrient	Sex	Studies (n)	Pooled (n)	Unit	EAR	Mean	SD	Percentage below EAR^*^	95% CI
Iron	W M	10 10	1314 572	mg/d	5 6	6.5 10.8	2.7 11.9	9 10	7–11 8–12
Selenium	W M	4 4	562 236	μg/d	45 45	54.4 72	19.1 23.5	44 27	40–48 21–33
Zinc	W M	9 9	1056 423	mg/d	6.8 9.4	7 8.5	2.2 2.6	50 66	47–53 61–71
Iodine	W M	2 2	338 110	μg/d	95 95	57.7 68.4	28.3 31.1	78 67	73–82 58–76
Copper	W M	2 2	486 170	mg/d	0.7 0.7	0.98 0.99	0.4 0.17	27 25	23–31 18–32
Molybdenum	W M	- -	- -	μg/d	34 34	-	-	-	-
Manganese	W M	-	-	mg/d	1.8 + 2.3 +	-	-	-	-
Chromium	W M	-	-	μg/d	20 + 34 +	-	-	-	-

EAR = Estimated Average Requirement, CI = Confidence Interval, += Adequate Intake, not enable to apply EAR cut-point method, *= Mean percentage of insufficient intake, calculated from EAR cut-point method,–Not available study.

## Supplementary Materials

The following are available online at https://www.mdpi.com/2072-6643/12/4/1072/s1, Figure S1: PRISMA (Preferred Reporting Items for Systematic Reviews and Meta-Analyses) flowchart for included and excluded studies in the article, Figure S2: The percentage of males and females at risk of deficiency for iron, zinc, selenium iodine and copper living in the community (A) or in institutions (B), Figure S3: Funnel plots showing the proportion of the population which were deficient against participant number in community dwelling individuals, Figure S4: Funnel plots showing the proportion of the population which were deficient against participant number in individuals living in institutions, Figure S5: Hazard ratios comparing level of deficiency for females (A) and males (B) between community and institution settings, Table S1: Outline of the combined quality assessment, Table S2: Summary of the study quality of the included studies.
