# Supplementary material for: Trace Mineral Intake and Deficiencies in Older Adults Living in the Community and Institutions: A Systematic Review
Source: Nutrients. 2020 Apr 13;12(4):1072. doi: 10.3390/nu12041072 (PMC7230219; doi:10.3390/nu12041072)
Supplement: Supplementary file 1 [file nutrients-12-01072-s001.pdf]

SUPPLEMENTARY MATERIAL

LITERATURE SEARCH:

The following search terms were used in PUBMED, EMBASE and Web of Science: ('senior' OR 'older person' OR 'older people' OR 'older adults' OR 'elderly' OR 'ageing' OR 'geriatric' OR 'advanced age') AND ('observational study' OR 'epidemiological study' OR 'population-based study' OR 'cross-sectional study' OR 'longitudinal study' OR 'prospective study' OR 'cohort study' OR 'cohort design' OR 'cross-sectional design' OR 'longitudinal design' OR 'population-based design' OR 'epidemiological design' OR 'prospective design') AND ('free living' OR 'independent living' OR 'community dwelling' OR 'non-institutionalised' OR 'institutionalised' OR 'care home' OR 'nursing home' OR 'retired home' OR 'dependent living' OR 'care centre') AND ('mineral intake' OR 'mineral deficiency' OR 'mineral inadequacy' OR 'nutrient intake' OR 'nutritional intake' OR 'nutrient deficient' OR 'nutrient deficiency' OR 'nutrient deficiencies' OR 'dietary intake' OR 'food intake' OR 'diet records' OR 'dietary adequacy' OR 'nutrition assessment' OR 'nutritional status' OR 'trace mineral' OR 'trace element').

QUALITY ASSESSMENT:

Supplementary Table 1: Outline of the combined quality assessment.

Component	Assessment Criteria	Points
Predefined study population (e.g. area, sample size, random-sampling or non-random sampling, inclusion period)	Information given	1
	No information	0
Inclusion and exclusion criteria	Clearly defined	1
	Not defined	0
	SELECTION	
A validated method as stated by FAO [1]	The method as outlined by FAO and expression of validation	2
(validated diet records (three days or more), food frequency questionnaire (FFQ), dietary history, 24-h dietary recall)	If the method outlined by FAO is used but no expression of validation	1
	Other methods or no information about the method	0

Selective reporting bias	All subjects completed the study (Reported data is equal to the initial sample size)	1
	Some subjects were lost but the description provided of those lost (Reported data is not equal to initial sample size but rationale is given)	1
	Some subjects were lost and no description of those lost (Reported data is not equal to initial sample size, no information or incomplete justification is given)	0
	OUTCOME	

Adapted by ter Borg et al. [2])

FAO: Food and Agriculture Organization of the United Nations [1]

Quality Score: 0 – 2 = Low quality, 3 – 4 = Moderate quality, 5 = High quality

Supplementary Table 2: Summary of the study quality of the included studies.

Reference	Selection bias		Outcome bias		Quality
	Predefined population	In/Exclusion criteria	Validated method	Transparent reporting	
(Wyka et al. [3])	1	0	1	1	Moderate
(Zhu et al. [4])	1	1	2	1	High
(Jiménez-Redondo et al. [5])	1	1	1	1	Moderate
(Engelheart and Akner [6])	1	1	1	1	Moderate
(Dumartheray et al. [7])	1	0	2	1	Moderate
(Li et al. [8])	1	1	1	1	Moderate
	1	0	1	1	Moderate

(Destefani et al. [9])					
	1	1	1	1	Moderate
(Feart et al. [10])					
	1	1	2	1	High
(Ocke et al. [11])	1	1	1	0	Moderate
(Sette et al. [12])	1	0	1	1	Moderate
(Biró et al. [13])	1	1	1	0	Moderate
(National Diet and Nutrition Survey [14])	1	1	2	1	High
	1	1	1	1	Moderate
USDA et al. [15])	1	1	1	1	Moderate
(NANS [16])					
	1	0	1	1	Moderate
(González et al. [17])					
	1	1	2	1	High
(Rakıcıoğlu et al. [18])					
(Fernández-Barrés et al. [19])	1	1	1	1	Moderate
	1	0	1	1	Moderate
(Woods et al. [20])					
(Iuliano et al. [21])	1	1	1	1	Moderate
(Lengyel et al. [22])	0	1	2	1	Moderate
(Lopez-Contreras et al. [23])	1	0	1	1	Moderate
	1	1	1	1	Moderate
(Leslie et al. [24])	1	1	1	1	Moderate

(Aghdassi et al. [25])	0	1	1	1	Moderate
(Rodríguez-Rejón et al. [26])	1	0	1	1	Moderate
(Assis et al. [27])	1	1	1	0	Moderate
(Martínez Tomé et al. [28])					
(Roussel et al. [29])					

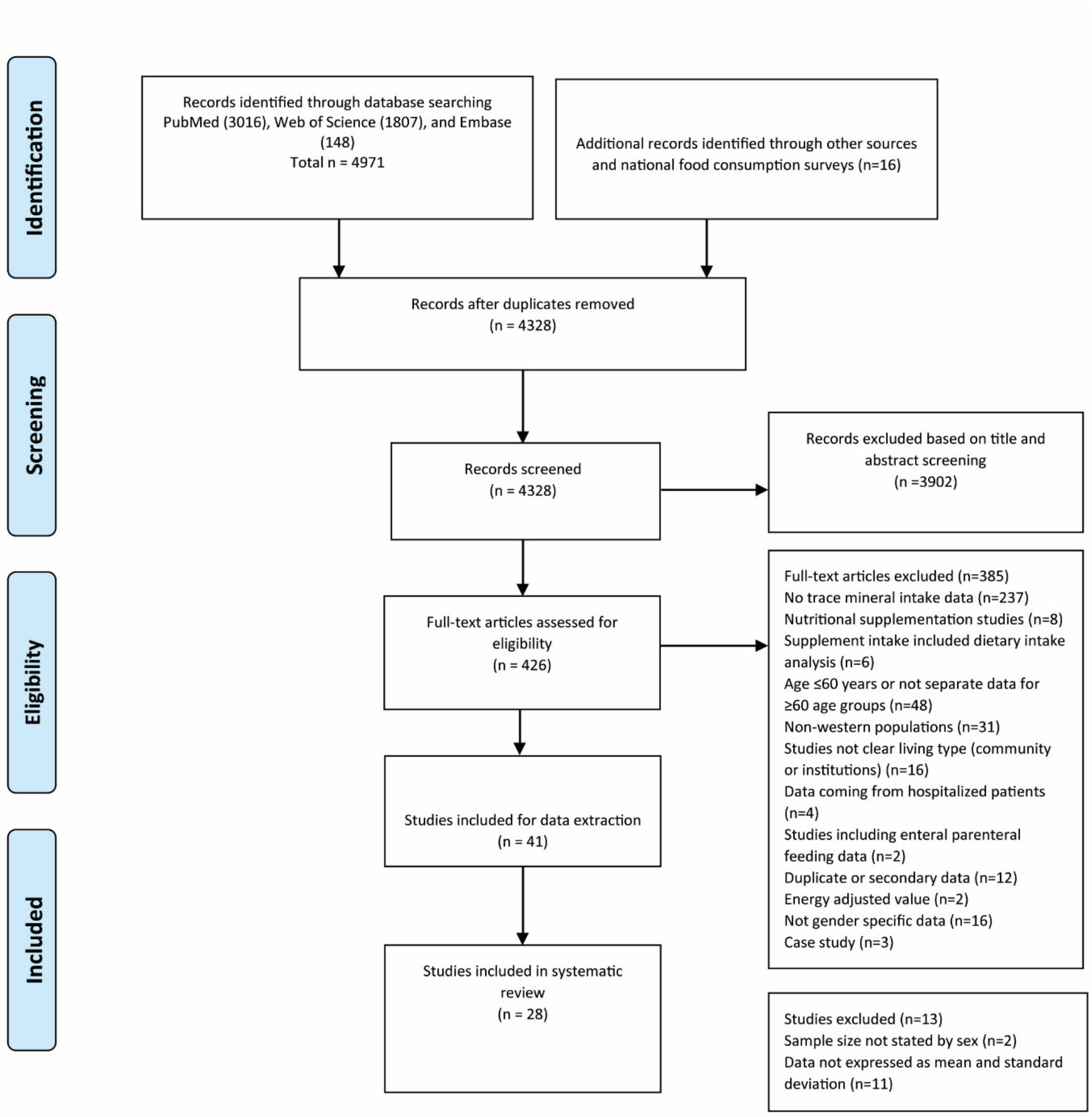

Supplementary Figure 1: PRISMA (Preferred Reporting Items for Systematic Reviews and Meta-Analyses) flowchart for included and excluded studies in the article.

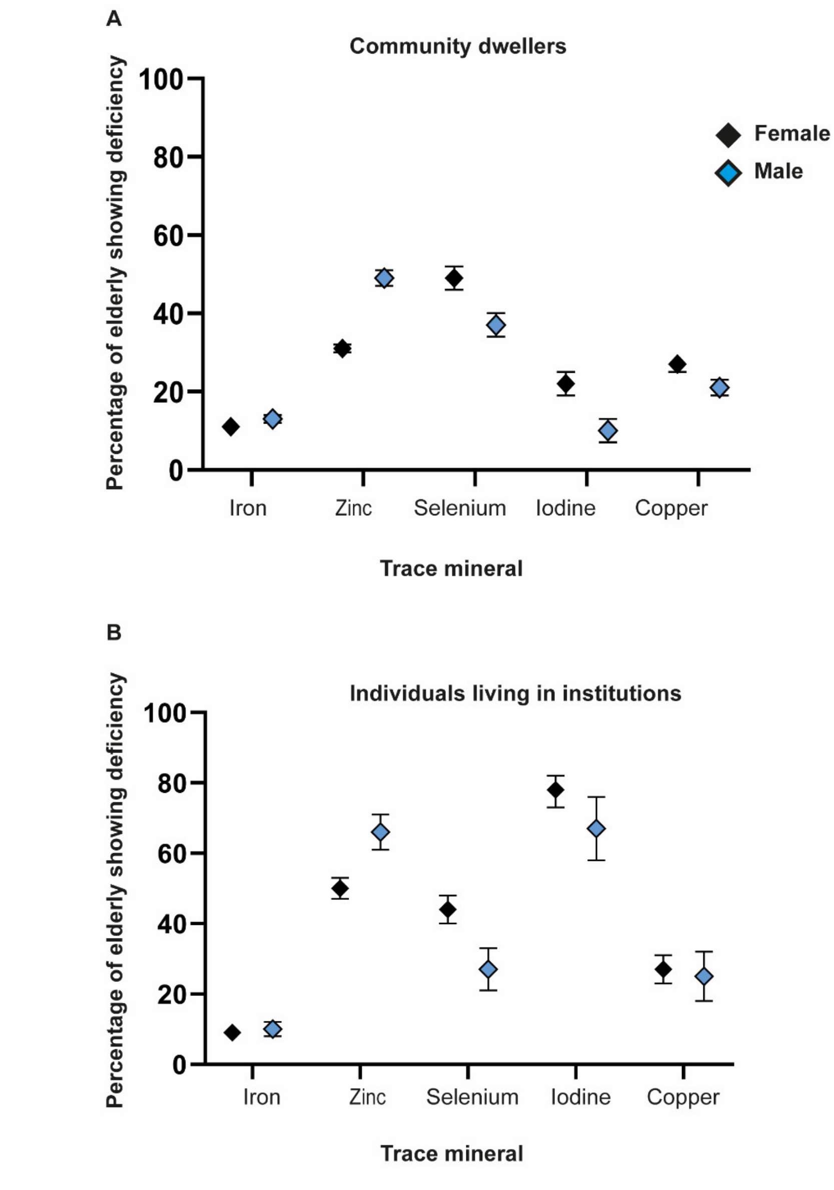

Supplementary Figure 2: The percentage of males and females at risk of deficiency for iron, zinc, selenium iodine and copper living in the community (A) or in institutions (B). Data are presented as mean percentage of population at risk of deficiency ± upper and lower limits.

Community dwelling participants.

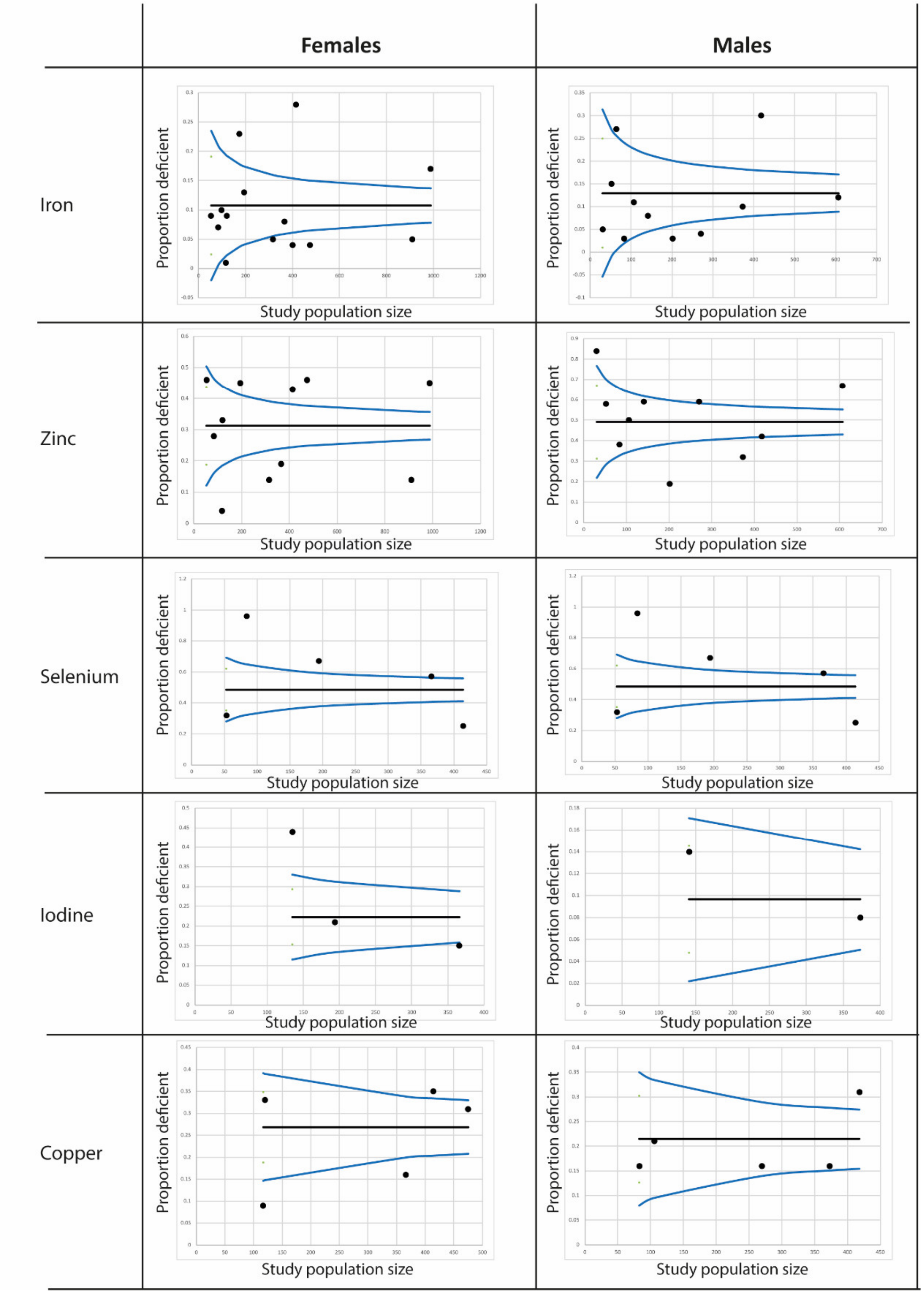

Supplementary Figure 3. Funnel plots showing the proportion of the population which were deficient against participant number in community dwelling individuals. Confidence limits (99.7% are indicated by the blue line).

Institution dwelling participants.

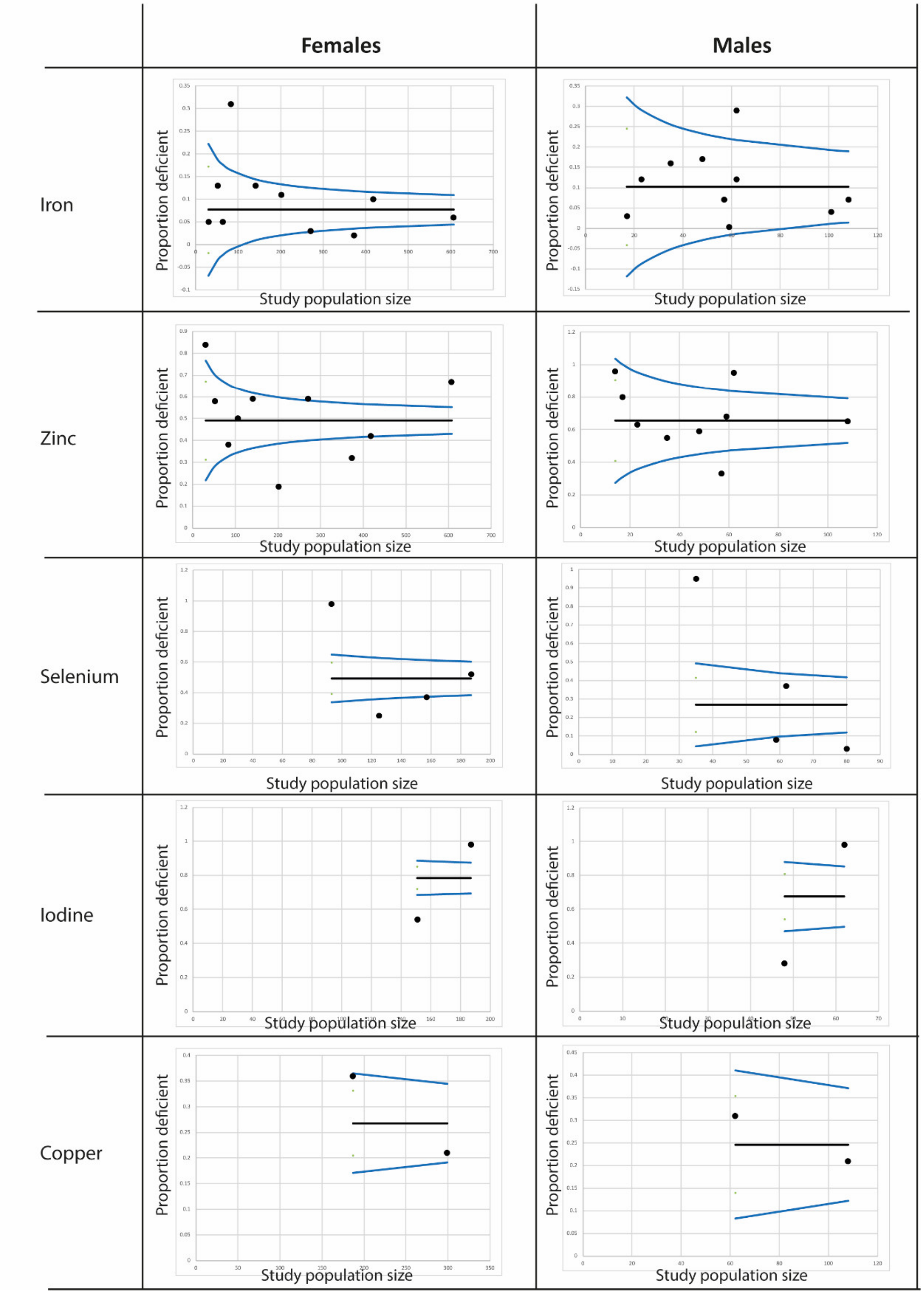

Supplementary Figure 4. Funnel plots showing the proportion of the population which were deficient against participant number in individuals living in institutions. Confidence limits (99.7% are indicated by the blue line).

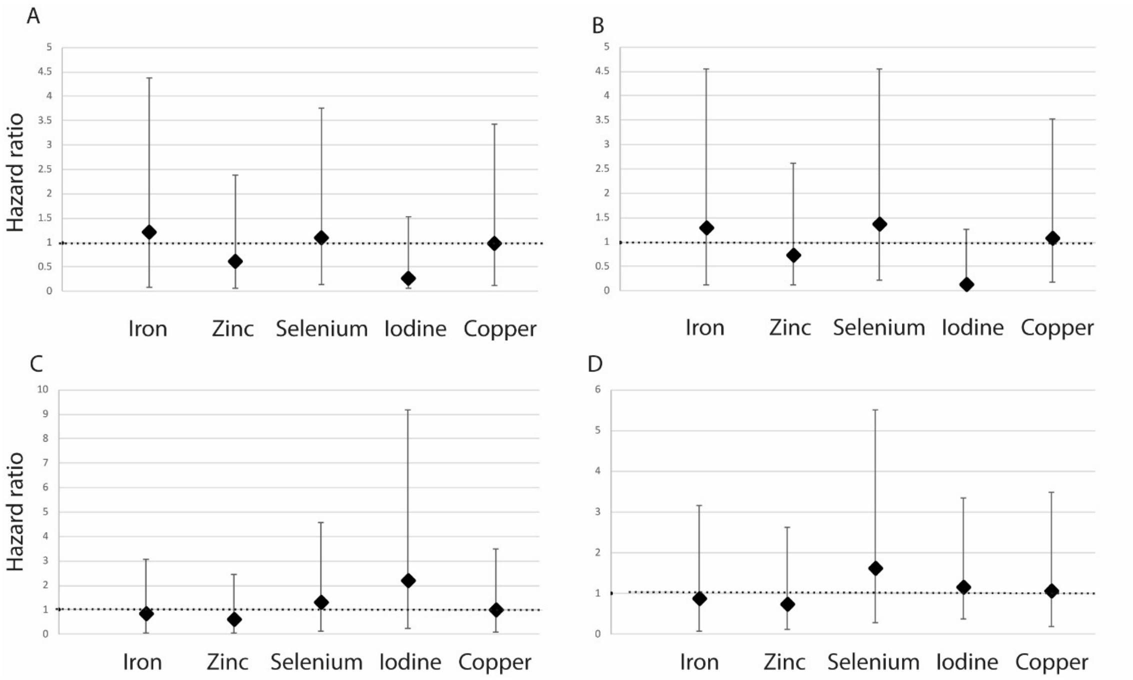

Supplementary Figure 5. A & B: Hazard ratios comparing level of deficiency for females (A) and males (B) between community and institution settings (B). C & D: Hazard ratios comparing level of deficiency between females and males for those in the community (C) and those living in institutions (D).

References

1. FAO. *Dietary Assessment: A Resource Guide to Method Selection and Application in Low Resource Settings*; FAO: Rome, Italy, 2018.
2. Ter Borg, S.; Verlaan, S.; Hemsworth, J.; Mijnarends, D.M.; Schols, J.M.G.A.; Luiking, Y.C.; de Groot, L.C.P.G.M. Micronutrient intakes and potential inadequacies of community-dwelling older adults: A systematic review. *Br. J. Nutr.* **2015**, *113*, 1195–1206, doi:10.1017/S0007114515000203.
3. Wyka, J.; Biernat, J.; Mikołajczak, J.; Piotrowska, E. Assessment of dietary intake and nutritional status (MNA) in Polish free-living elderly people from rural environments. *Arch. Gerontol. Geriatr.* **2012**, *54*, 44–49, doi:10.1016/j.archger.2011.02.001.
4. Zhu, K.; Devine, A.; Suleska, A.; Tan, C.Y.; Toh, C.Z.J.; Kerr, D.; Prince, R.L. Adequacy and change in nutrient and food intakes with aging in a seven-year cohort study in elderly women. *J. Nutr. Health Aging* **2010**, *14*, 723–729.
5. Jiménez-Redondo, S.; Beltrán de Miguel, B.; Gavidia Banegas, J.; Guzmán Mercedes, L.; Gómez-Pavón, J.; Cuadrado Vives, C. Influence of nutritional status on health-related quality of life of non-institutionalized older people. *J. Nutr. Health Aging* **2014**, *18*, 359–364, doi:10.1007/s12603-013-0416-x.
6. Engelheart, S.; Akner, G. Dietary intake of energy, nutrients and water in elderly people living at home or in nursing home. *J. Nutr. Health Aging* **2015**, *19*, 265–272, doi:10.1007/s12603-015-0440-0.
7. Dumartheray, E.W.; Krieg, M.-A.; Cornuz, J.; Whittamore, D.R.; Lanham-New, S.A.; Burckhardt, P. Energy and nutrient intake of Swiss women aged 75–87 years. *J. Hum. Nutr. Diet.* **2006**, *19*, 431–435, doi:10.1111/j.1365-277X.2006.00729.x.

8. Li, W.; Youssef, G.; Procter-Gray, E.; Olendzki, B.; Cornish, T.; Hayes, R.; Churchill, L.; Kane, K.; Brown, K.; Magee, M.F. Racial Differences in Eating Patterns and Food Purchasing Behaviors among Urban Older Women. *J. Nutr. Health Aging* **2017**, *21*, 1190–1199, doi:10.1007/s12603-016-0834-7.
9. Destefani, S.A.; Corrente, J.E.; Paiva, S.A.R.; da Silva Mazeto, G.M.F. Prevalence of iodine intake inadequacy in elderly Brazilian women. A cross-sectional study. *J. Nutr. Health Aging* **2015**, *19*, 137–140, doi:10.1007/s12603-014-0526-0.
10. Feart, C.; Alles, B.; Merle, B.; Samieri, C.; Barberger-Gateau, P. Adherence to a Mediterranean diet and energy, macro-, and micronutrient intakes in older persons. *J. Physiol. Biochem.* **2012**, *68*, 691–700, doi:10.1007/s13105-012-0190-y.
11. Ocke, M.C.; Buurma-Rethans, E. J. M.; De Boer, E. J.; Wilson-Van den Hooven, C.; Etemad-Ghameslou, Z.; Drijvers, J. J. M. M.; Van Rossum, C. T. M. *Diet of Community-Dwelling Older Adults; Dutch national food consumption survey older adults 2010-2012.* National Institute for Public Health and the Environment Ministry of Health, Welfare and Sport: Netherlands, 2013; Volume 127.
12. Sette, S.; Le Donne, C.; Piccinelli, R.; Arcella, D.; Turrini, A.; Leclercq, C. INRAN-SCAI 2005-6 Study Group. The third Italian National Food Consumption Survey, INRAN-SCAI 2005-06—Part 1: Nutrient intakes in Italy. *Nutr. Metab. Cardiovasc. Dis.* **2011**, *21*, 922–932, doi:10.1016/j.numecd.2010.03.001.
13. Biró, L.; Szeitz-Szabó, M.; Biró, G.; Sali, J. Dietary survey in Hungary, 2009. Part II: Vitamins, macro- and microelements, food supplements and food allergy. *Acta Aliment.* **2011**, *40*, 301–312, doi:10.1556/AAlim.40.2011.2.14.
14. National Diet and Nutrition Survey (NDNS). Results from Years 7 and 8 (Combined). 2018. Available online: https://www.gov.uk/government/statistics/ndns-results-from-years-7-and-8-combined (accessed on 17 March 2019).
15. U.S. Department of Agriculture, Agricultural Research Service. Nutrient intakes from Food: Mean Amounts Consumed Per Individual, By Gender and Age, What We Eat in AMERICA, NHANES 2019. 2015–2016. Available online: http://www.ars.usda.gov/ba/bhnrc/fsrg (accessed on 26 March 2019).
16. National Adult Nutrition Survey (NANS) 2008-2010. Available online: http://www.iuna.net (accessed on 28 March 2019).
17. González, S.; Huerta, J.M.; Fernández, S.; Patterson, E.M.; Lasheras, C. Food intake and serum selenium concentration in elderly people. *Ann. Nutr. Metab.* **2006**, *50*, 126–131, doi:10.1159/000090633.
18. Rakıcıoğlu, N.; Aksoy, B.; Tamer, F.; Yıldız, E.A.; Samur, G.; Pekcan, G.; Besler, H.T. Nutritional status and eating habits of the institutionalised elderly in Turkey: A follow-up study. *J. Hum. Nutr. Diet.* **2016**, *29*, 185–195, doi:10.1111/jhn.12320.
19. Fernández-Barrés, S.; Martín, N.; Canela, T.; García-Barco, M.; Basora, J.; Arija, V.; Project Atdom-Nut Group. Dietary intake in the dependent elderly: Evaluation of the risk of nutritional deficit. *J. Hum. Nutr. Diet.* **2016**, *29*, 174–184, doi:10.1111/jhn.12310.
20. Woods, J.L.; Walker, K.Z.; Iuliano Burns, S.; Strauss, B.J. Malnutrition on the menu: Nutritional status of institutionalised elderly Australians in low-level care. *J. Nutr. Health Aging* **2009**, *13*, 693–698.
21. Iuliano, S.; Olden, A.; Woods, J. Meeting the nutritional needs of elderly residents in aged-care: Are we doing enough? *J. Nutr. Health Aging* **2013**, *17*, 503–508, doi:10.1007/s12603-013-0042-7.
22. Lengyel, C.O.; Whiting, S.J.; Zello, G.A. Nutrient inadequacies among elderly residents of long-term care facilities. *Can. J. Diet. Pract. Res.* **2008**, *69*, 82–88, doi:10.3148/69.2.2008.82.
23. Lopez-Contreras, M.J.; Zamora-Portero, S.; Lopez, M.A.; Marin, J.F.; Zamora, S.; Perez-Llamas, F. Dietary intake and iron status of institutionalized elderly people: Relationship with different factors. *J. Nutr. Health Aging* **2010**, *14*, 816–821.
24. Leslie, W.S.; Lean, M.E.J.; Woodward, M.; Wallace, F.A.; Hankey, C.R. Unidentified under-nutrition: Dietary intake and anthropometric indices in a residential care home population. *J. Hum. Nutr. Diet.* **2006**, *19*, 343–347, doi:10.1111/j.1365-277X.2006.00719.x.
25. Aghdassi, E.; McArthur, M.; Liu, B.; McGeer, A.; Simor, A.; Allard, J.P. Dietary intake of elderly living in Toronto long-term care facilities: Comparison to the dietary reference intake. *Rejuvenation Res.* **2007**, *10*, 301–309, doi:10.1089/rej.2006.0530.
26. Rodríguez-Rejón, A.I.; Ruiz-López, M.D.; Artacho, R. Dietary Intake and Associated Factors in Long-Term Care Homes in Southeast Spain. *Nutrients* **2019**, *11*, doi:10.3390/nu11020266.

27. Assis, B.S.; Jairza, J.M.B.-M.; Lopes, J.A.; Roriz, A.K.C.; Melo, A.L.; Previdell, A.; Aquino, R.D.C.; Ramos, L.B. Micronutrient intake in elderly living in nursing homes. *Nutr. Hosp.* **2018**, *35*, 59–64, doi:10.20960/nh.1348.
28. Martínez Tomé, M.J.; Rodríguez, A.; Jiménez, A.M.; Mariscal, M.; Murcia, M.A.; García-Diz, L. Food habits and nutritional status of elderly people living in a Spanish Mediterranean city. *Nutr. Hosp.* **2011**, *26*, 1175–1182, doi:10.1590/S0212-16112011000500038.
29. Roussel, A.-M.; Andriollo-Sanchez, M.; Ferry, M.; Bryden, N.A.; Anderson, R.A. Food chromium content, dietary chromium intake and related biological variables in French free-living elderly. *Br. J. Nutr.* **2007**, *98*, 326–331, doi:10.1017/S000711450770168X.

© 2020 by the authors. Submitted for possible open access publication under the terms and conditions of the Creative Commons Attribution (CC BY) license (http://creativecommons.org/licenses/by/4.0/).
